# From believing to behaving: Unpacking teacher emotion as the mediator between information literacy self-efficacy and information-empowered teaching engagement

**DOI:** 10.3389/fpsyg.2025.1696627

**Published:** 2026-01-07

**Authors:** Xinfeng Xie, Chuyi Du, Jing Cheng, Haiyan Zhang

**Affiliations:** 1 School of Foreign Languages, Jimei University, Xiamen, China; 2 School of Arts and Sciences, Fujian Medical University, Fuzhou, China

**Keywords:** information literacy, parallel mediation, self-efficacy, teacher emotions, teaching engagement

## Abstract

**Introduction:**

In the era of artificial intelligence, information literacy is a crucial skill for teachers, enabling the effective integration of technology into pedagogy. This study examines the psychological factors influencing the information-empowered teaching engagement of university English teachers.

**Methods:**

A cross-sectional survey was conducted with 224 university English teachers in Southeast China. Data were collected using a composite questionnaire measuring information literacy self-efficacy, teacher emotion (both positive and negative), and information-empowered teaching engagement. The data were analyzed via SmartPLS to test the proposed mediation model.

**Results:**

The findings indicate that university English teachers experience a mix of positive and negative emotions when applying information technology. Information literacy self-efficacy was identified as a primary factor promoting teaching engagement. Both positive and negative emotions were found to play substantial and parallel mediating roles between self-efficacy and engagement.

**Discussion:**

The study highlights the complex emotional landscape accompanying technology integration. It proposes the design of hybrid professional development programs for technology-rich environments. These programs should concurrently provide emotional support and technical training to enhance teachers’ information literacy self-efficacy and, consequently, their information-empowered teaching engagement.

## 1 Introduction

The advancement in information technology represented by Large Language Models (LLM) and artificial intelligence (AI) in recent years has changed the way of acquiring knowledge (Cohen et al., 2020). AI tools tend to present knowledge in an authoritative way, yet whether and how much we can trust the information provided remain questionable. It depends on the users’ accurate evaluation of the information, which is fundamentally about information literacy. Information literacy refers to the ability to understand, construct, and create new knowledge, as well as to discover, analyze, and solve problems, including components like information awareness, information knowledge, information application skills, and information ethics and security (Ministry of Education of the People’s Republic of China, 2021). The critical importance of these competencies is powerfully illustrated in specialized fields such as engineering education, where critical thinking, data analysis, and ethical decision-making, ensuring reliability and rigor in professional practice are underpinned (Tripon, 2025). The rapid development of AI tools gives rise to higher requirements for information literacy (Chen et al., 2023). In this regard, information literacy has emerged as one of the essential competences for language teachers in the context of educational informatization, enabling them to effectively use the vast resources offered by information technologies.

Language teachers’ information literacy, or digital literacy, a similar construct gaining winds at the very recent years due to the upcoming of the AI era, is always one of the popular topic in the study of applied linguistics. The construct itself and its essential components have been discussed in the existing body of research (Wang, 2022; Qin and He, 2009). Liu et al. (2017) explore the landscape of information literacy and discuss the potential path for its enhancement from a technical standpoint. However, information literacy is not about the possession of skills only. Researchers argue that it should be about the appreciation of information as well (Bruce, 1997), and the importance of psychological factors like self-efficacy and academic motivation in the development of information literacy skills should be acknowledged (Diliello et al., 2011; Ross et al., 2016). These findings underscore the fact that information literacy is multifaceted, and that psychological aspects are as critical as theoretical and technical components.

Even though information literacy has been extensively studied, the psychological dimension of it is still neglected by researchers. In addition, although existing studies have discussed teachers’ attitude toward (e.g., Yang et al., 2021) and emotion about (e.g., Azzaro and Martínez Agudo, 2018) information technology, the discussion about the relationship between these two psychological aspects and teachers’ teaching engagement is few. Therefore, this study intends to address this gap by focusing on the relationship between information literacy self-efficacy, teacher emotion, and information-empowered teaching engagement. Special attention will be paid to examine self-efficacy perceived and emotions experienced by university English teachers in applying information technology to their teaching.

## 2 Literature review

### 2.1 Information literacy

With the development of modern educational technology, the professional development of English teachers requires continuous improvement of professional knowledge, teaching skills and professional attitude through various means in the information technology environment (Jiao et al., 2009; Motteram and Dawson, 2025). Therefore, educational informatization requires English teachers not only to possess the “information teaching literacy” that combines information literacy with teaching ability (Chen, 2010; Jiang, 2024) and also maintain a positive emotional attitude toward information technology (Azzaro and Martínez Agudo, 2018; Beaudry and Pinsonneault, 2010; Wong et al., 2013). Correspondingly, Jia and Zhang (2023) proposed that information is a comprehensive concept emphasizing the characteristics of the times, while literacy emphasizes the emotions and values formed through acquired experience. This viewpoint offers the latest interpretation of information literacy that it not only encompasses the dimension of technical capabilities but also the dimension of emotions, emphasizing the comprehensiveness of information literacy.

However, existing research primarily discusses the definition, current status, enhancement strategies, evaluation criteria, and influencing factors of information literacy from the perspectives of traditional technological development and teaching capability enhancement (Wang and Bu, 2022). Although some studies have explored topics related to psychological constructs such as teacher self-efficacy in technological environments (Drossel et al., 2017; Garzon and Garzon, 2023) and teacher identity (Hanell, 2017; Zhang et al., 2023; Su, 2023), there remains a lack of micro-empirical research on the psychological aspect of information literacy, which deserves the attention of the academic community.

### 2.2 Teaching engagement, self-efficacy, and teacher emotion

Teaching engagement indicates the extent to which teachers commit themselves physically and mentally in teaching practice. It encompasses not only the explicit investment of teachers’ time and energy but also the implicit investment of their experiences and emotions (Kirkpatrick and Johnson, 2014; Mou et al., 2020). In the contemporary era of AI, this concept has evolved to encompass more specialized forms of practice. Specifically, we propose that information-empowered teaching engagement refers to a professional practice process in which teachers, within information-based teaching environments, proactively utilize informational data, tools, and digital resources to carry out teaching across the cognitive, behavioral, and emotional dimensions. This evolution aligns with existing frameworks, such as that of Klassen et al. (2013), who formulated a framework of teacher engagement including physical, cognitive, emotional and social engagement.

Such classifications indicate the inherent relationship between teaching engagement and teacher emotion. Khammat (2022) reports that people who are deeply engaged in their job are more likely to demonstrate better performance, and greater resilience (a positive emotion determining the ability to sustain a long-term career through the challenges of the profession). Similarly, higher job engagement can generate stable and dedicated positive emotional experiences or emotional states (Hemami, 2024; Inceoglu and Warr, 2011). Empirical studies have also shown a strong correlation between teachers’ work engagement and their professional wellbeing. Work engagement represents a positive mental state (Qu et al., 2025), and high levels of engagement is beneficial to fostering teachers’ psychological health (Greenier et al., 2021). However, there is a relative scarcity of research regarding the influence of teacher emotion on their teaching engagement.

Self-efficacy is viewed as a person’s beliefs in their capacity to successfully fulfill certain task (Bandura, 1978). With regard to its correlation with teacher emotion, existing studies has centered on its influence on stress, a negative emotion which further affects burnout, job satisfaction and teacher attrition (Troesch and Bauer, 2017). However, the role of self-efficacy in predicting teacher emotions (positive ones and negative ones) as a hole has been understudied (Wang and Pan, 2023). As a construct of positive self-assessment, self-efficacy reflects an individual’s ability and perception to control and successfully influence the environment (Burić and Macuka, 2018; Hobfoll et al., 2003). Likewise, teacher self-efficacy, which refers to teachers’ beliefs about what they can do in terms of a particular teaching task or instructional context, has been shown to influence motivational and behavioral processes (Landrieu, 2024; Tschannen-Moran et al., 1998), especially when their teaching behaviors interact with personal and external impact factors (Wang et al., 2024). In short, previous studies have indicated that higher teacher self-efficacy correlates with superior teaching quality compared to those with lower self-efficacy (Hoy et al., 2009; Landrieu, 2024; Zee and Koomen, 2016), yet its correlation with emotion is not thoroughly studied.

With the topics in research on language teaching and learning shifting toward the emotional dimension (Martínez Agudo, 2018), language teacher emotion has gradually attracted scholars’ attention (Chen and Cheng, 2022). Early studies primarily focused on negative emotions experienced in English teaching, such as work stress, professional burnout, and anxiety, without considering teacher emotion as an important factor affecting teaching itself (Zembylas, 2003). Over the past decade, studies on language teacher emotion have mainly concentrated on the emotional experiences of language teachers in different teaching contexts (Xie and Jiang, 2023), and some have indicated a significant correlation between teacher self-efficacy and their emotions (Burić et al., 2020; Hascher and Hagenauer, 2016). Similarly, Lo (2023) posits emotion as a bridge that connects cognitive belief (such as self-efficacy and self-concept) to behavioral engagement. In terms of the relationship between language teacher emotion and teaching engagement, Cowie (2011) believes that language teacher emotion is an important variable affecting teaching behavior, an idea partially supported by Azari Noughabi et al. (2022) who argue that English teachers’ positive emotions influence their work engagement. Dilekçi et al. (2025) draw a similar conclusion that teachers’ positive emotions are critical antecedents and a strong predictor of their work engagement. Lo and Punzalan (2025) further explain that teachers participating in Positive Psychology (PP) interventions generally reported significant improvements in such positive emotions as optimism, which are directly related to higher work engagement, manifested as increased commitment to teaching tasks and active participation in professional development. Overall, despite the existing studies recognizing the connections from self-efficacy to emotion and onward to engagement, the exact mediating mechanism of emotions within this pathway remains a “black box,” necessitating further research.

In summary, the existing literature on teaching engagement, self-efficacy, and teacher emotion has to a certain extent informed us the interrelationship among them. Studies have also consistently highlighted the importance of self-efficacy in language teaching. However, several gaps remain in the current understanding of this topic. Specifically, there is scarce research on the pathway of self-efficacy’s influence on teaching engagement and the impact of teacher emotion on teaching engagement, which deserves a close examination.

## 3 Research methodology

### 3.1 Research questions

The study is anchored by three pivotal research questions designed to explore the psychological dynamics underpinning the engagement of university English teachers in information-empowered teaching environments:

1. What are the features of emotions experienced by university English teachers in the information-empowered teaching context?
2. What is the relationship between information literacy self-efficacy, teacher emotion, and information-empowered teaching engagement?
3. Do positive and negative emotions mediate the relationship between university English teachers’ information literacy self-efficacy and information-empowered teaching engagement? If so, what is the magnitude and significance of the mediating effect?

### 3.2 Subjects

This study employed a convenience sampling method to recruit subjects from universities of various levels in Southeast China. A total of 224 university English teachers participated in the project voluntarily and filled out the questionnaire, which was distributed to them through Wenjuanxing,^1^ a popular online questionnaire website, in October 2024. The questionnaire link was sent out via faculty WeChat groups, professional networks of the research team, and academic social media communities specializing in English language teaching. The online survey platform mandated responses to all items, thus eliminating missing data. Subsequently, all submissions were manually screened by the researchers to disqualify eight invalid responses, resulting in a final sample of 224 valid questionnaires and an effective response rate of 96.6%.

Table 1 shows the demographic characteristics of the final sample. There were 151 female teachers (67%) and 73 male teachers (33%), a distribution that aligns with the general gender composition observed in the field of English language teaching in Chinese universities. In terms of the age, the participants fell into four age groups, with the majority of them aged 31–40 and 41–50, collectively constituting 76% of the sample. This indicates that the data primarily reflects the perspectives of teachers who were in the core stages of their academic careers. Regarding institutional representation, the sample achieved a relatively balanced distribution across National Key Universities (31%), Provincial Key Universities (28%), and Local Colleges (41%). This distribution helps to capture teaching experiences from varying academic environments and resource levels. At last, the sample included Lecturers (43%), Associate Professors (41%), and Professors (16%), mirroring the pyramidal structure typical of the academic hierarchy in Chinese higher education.

**Table 1:** Demographic information of the subjects (*N* = 224).

Variable	Gender		Age				School type			Academic title		
	M	F	≤30	31–40	41–50	51–60	National Key University	Provincial Key University	Local college	Professor	Associate professor	Lecturer
Quantity	73	151	5	63	108	48	70	62	92	35	92	97
Percentage	33%	67%	2%	28%	48%	22%	31%	28%	41%	16%	41%	43%

### 3.3 Instruments

Drawing on the research by Yang et al. (2021), the *Composite Questionnaire of Information Literacy for University English Teachers* (see Supplementary Appendix 1) was developed. The three scales included in this composite questionnaire were developed through a rigorous process of adaptation and contextualization to suit the specific research context. During the development phase, a pilot study was conducted with 30 university English teachers who were not included in the main survey sample. These teachers were also asked to offer constructive feedback on the content of the questionnaire. With their insights, particularly regarding the clarity and comprehensibility of the language used in the questionnaire, the items were refined to ensure that they were both precise in wording and easily understood, thereby enhancing the overall quality and effectiveness of the questionnaire.

The questionnaire consists of three independent scales, which are specifically introduced as follows:

1. University English Teacher Emotion Scale. Given the lack of well-established scale for measuring teacher emotions in information technology-related contexts, most frequently reported emotions from existing literature (Azzaro and Martínez Agudo, 2018; Xie and Jiang, 2023) were chosen to develop this scale. All statements were well written after referring to the literature and consulting to one expert specialized in studying emotions. The scale is a 5-point Likert scale, where 1 = Strongly Disagree; 2 = Disagree; 3 = Neutral; 4 = Agree; 5 = Strongly Agree. It contains 7 items, with 3 items assessing positive emotions and 4 items assessing negative emotions. As is shown in Table 2, both emotion sub-scales demonstrated strong psychometric properties. Positive Emotion showed excellent reliability (*α* = 0.877, CR = 0.924) and convergent validity (AVE = 0.802), while Negative Emotion also showed good reliability (α = 0.845, CR = 0.895) and convergent validity (AVE = 0.680), with all metrics exceeding their respective thresholds (Hair et al., 2022).
2. Information Literacy Self-Efficacy Scale. It was developed based on the existing literature, primarily drawing on the 13-item scale translated and validated by Atikuzzaman and Zabed Ahmed (2023). The original 13-item scale was adapted through a process of systematic translation, back-translation, and cultural adaptation. First, one researcher produced an initial translation, which was then reviewed by a subject specialist to align with the context of university English teaching in China. Second, back-translation and multiple rounds of revision were conducted to ensure conceptual consistency. A specific example is the transformation of the original item “Select information most appropriate to the information need” into “I can screen out important information from online resources based on teaching needs,” where back-translation confirmed conceptual equivalence while successfully contextualizing it for the English teaching environment. Third, culturally irrelevant items, such as using traditional library catalogs, were removed, and the focus was narrowed from general information literacy to the acquisition and evaluation of digital teaching resources. For instance, “Create bibliographic records and organize the bibliography” was replaced with “I can integrate existing and new information to expand the English teaching resource database” (see Supplementary Appendices 1, 2) The final scale emerged as an 8-item contextually refined 5-point Likert scale, where 1 = Strongly Disagree; 2 = Disagree; 3 = Neutral; 4 = Agree; 5 = Strongly Agree. It showed excellent reliability (*α* = 0.930, CR = 0.942) and good convergent validity (AVE = 0.671), with all values exceeding their thresholds (see Table 2).
3. Information-Empowered Teaching Engagement Scale. It was primarily derived from Yang et al. (2021). To achieve a more focused measure, the ‘belief in curriculum standard’ dimension from the original instrument was removed and the retained items were reorganized and refined to form a cohesive scale. This scale is a 5-point Likert scale, where 1 = Strongly Disagree; 2 = Disagree; 3 = Neutral; 4 = Agree; 5 = Strongly Agree. It contains 12 items, including three dimensions, namely emotional engagement (3 items), behavioral engagement (5 items), and cognitive engagement (4 items). Table 2 indicated that the scale demonstrated excellent reliability (α = 0.956, CR = 0.972) and convergent validity (AVE = 0.920).

**Table 2:** Structural reliability and validity verification.

Dimension	Cronbach’s alpha	Overall reliability (rho_a)	Overall reliability (rho_c)	Mean variance extraction (AVE)
Negative emotion	0.845	0.865	0.895	0.680
Positive emotion	0.877	0.877	0.924	0.802
Self-efficacy	0.930	0.931	0.942	0.671
Teaching engagement	0.956	0.958	0.972	0.920

SmartPLS was employed to perform exploratory factor analysis (EFA). By using principal component analysis, EFA extracted four factors with eigenvalues greater than 1, and the first factor accounted for 38.949% of the total variance. As shown in Table 3, these four factors collectively accounted for 74.130% of the total variance. After varimax rotation, the variances of 4 components were redistributed as 29.749, 15.580, 15.149, and 13.391%, respectively. This clear and interpretable factor structure demonstrated the sound construct validity of the composite questionnaire.

**Table 3:** Results of EFA: total variance explained by initial and rotated factors.

Component	Initial eigenvalues			Extraction sums of squared loadings			Rotation sums of squared loadings		
	Total	% of variance	Cumulative %	Total	% of variance	Cumulative %	Total	% of variance	Cumulative %
1	7.011	38.949	38.949	7.011	38.949	38.949	5.380	29.889	29.889
2	2.557	14.207	53.156	2.557	14.207	53.156	2.792	15.514	45.403
3	2.403	13.347	66.504	2.403	13.347	66.504	2.716	15.086	60.489
4	1.373	7.626	74.130	1.373	7.626	74.130	2.455	13.640	74.130
5	0.527	2.929	77.059						
6	0.495	2.750	79.809						
7	0.463	2.570	82.379						
8	0.431	2.395	84.774						
9	0.411	2.285	87.059						
10	0.379	2.104	89.163						
11	0.353	1.964	91.126						
12	0.319	1.772	92.899						
13	0.312	1.731	94.629						
14	0.290	1.610	96.239						
15	0.242	1.343	97.582						
16	0.217	1.206	98.789						
17	0.120	0.667	99.455						
18	0.098	0.545	100.000						

Factor analysis was conducted to examine the cross-loadings of each measurement item on its designated and non-designated factors. The results indicated that all items demonstrated satisfactory discriminant validity. Specifically, each item loaded highly on its intended factor (with all absolute values exceeding 0.754, see Supplementary Appendix 1). For instance, factor loadings were as follows: teaching engagement (0.762–0.849), self-efficacy (0.754–0.842), negative emotions (0.774–0.832), and positive emotions (0.845–0.879). This distinct pattern of loadings confirmed that the latent factors were well differentiated, and the four constructs measured by the scale were independent and clearly distinct from one another.

The discriminant validity of the constructs was also tested by using the Heterotrait-Monotrait (HTMT) ratio of correlations. Table 4 shows that all HTMT values were substantially below 0.85, a conservative threshold suggested by Henseler et al. (2015), demonstrating that the constructs were empirically distinct from one another. Notably, the value between negative emotion and positive emotion was the lowest (0.073), while the highest value was observed between teaching engagement and self-efficacy (0.460). These results overtly showed the discriminant validity, confirming that the four constructs, despite being correlated, were distinct from each other, thereby robustly supporting the discriminant validity of the measurement model employed in this study.

**Table 4:** HTMT ratio for discriminant validity.

Variable	Negative emotion	Positive emotion	Self-efficacy	Teaching engagement
Negative emotion				
Positive emotion	0.073			
Self-efficacy	0.303	0.304		
Teaching engagement	0.391	0.402	0.460	

The KMO measure of sampling adequacy and Bartlett’s test of sphericity (see Table 5) were also examined. The results presented that the KMO measure was 0.897, and Bartlett’s test of sphericity was significant, with an approximate chi-square value of 2747.978 (*p* < 0.001). As a result, the composite questionnaire was suitable for factor analysis.

**Table 5:** KMO and Bartlett’s tests of sphericity.

KMO		0.897
Bartlett’s test of sphericity	Approximate Chi-square	2747.978
	*df*	153
	Sig.	0.000

### 3.4 Data collection and analysis

In addition to the aforementioned statistical remedies employed, procedural measures were also adopted in the data collection process to further mitigate the potential for common method bias. These measures, aligned with the guidelines of Podsakoff et al. (2003), included respondent anonymity, the use of reverse-coded items, and randomization of question order.

Following preliminary analyses that included descriptive statistics and correlation analysis, the hypothesized relationships and mediation mechanisms were examined within a structural equation modeling (SEM) framework. All analyses were carried out with SmartPLS 4 software. This analytical approach was chosen because it allowed for the simultaneous estimation of all direct and indirect paths while controlling for measurement error. The significance of the mediation effects was tested using the bias-corrected bootstrap method with 5,000 resamples.

## 4 Results

### 4.1 Emotional experiences of university English teachers in information-empowered teaching

To explore the emotional experiences of university English teachers in the process of information-empowered teaching, descriptive analysis and normality tests on the relevant variables were conducted.

According to Table 6, the mean score of positive emotions among university English teachers was 12.07, which fell within the higher end of the scale (12–15). This indicated that these teachers reported a level of positive emotions that exceeded 80% of the maximum score; conversely, the mean score of negative emotions was 10.55, placing it in the medium range (4–12), suggesting that the level of negative emotions experienced by university English teachers were lower than 60% of the maximum score (Shao et al., 2013). The results demonstrated the predominance of positive emotional responses of university English teachers toward the integration of information technology in their teaching practices.

**Table 6:** Levels of positive and negative emotions.

Variable	Score range	Mean	SD	Median	Mode	Minimum	Maximum	Skewness (standard error)	Kurtosis (standard error)
Positive emotion	3–15	12.07	2.32	12	12	3	15	−0.687 (0.163)	0.422 (0.324)
Negative emotion	4–20	10.55	3.34	11	10	4	25	0.184 (0.163)	0.044 (0.324)

Furthermore, this study also examined the relationship between demographic variables and teachers’ emotional experiences, conducting multiple regression analysis with gender, age, school type, and academic title as independent variables, and positive and negative emotions as dependent variables. The results showed that demographic variables did not have a statistically significant impact on university English teacher emotions in information-empowered teaching (*p* > 0.05).

### 4.2 Correlation between information literacy self-efficacy, teacher emotions, and engagement in information-empowered teaching

In order to examine the correlations among college English teachers’ information literacy self-efficacy, teacher emotion, and information-empowered teaching engagement, this study conducted a descriptive analysis before a Pearson correlation analysis.

As shown in Table 7, significant correlations between the information literacy self-efficacy of university English teachers and various aspects of their teaching experience were found. There was a positive relationship between the university English teachers’ self-efficacy and their positive emotion (*r* = 0.274, *p* < 0.001), as well as a substantial link with their teaching engagement (*r* = 0.435, *p* < 0.001). On the flip side, an inverse relationship was observed between their self-efficacy and negative emotions (*r* = −0.269, *p* < 0.001). These findings implied that as these teachers’ confidence in applying information technology in English teaching grows, their level of information-empowered teaching engagement also rises, accompanied by an increased likelihood of experiencing positive emotions and a reduced possibility of experiencing negative emotions.

**Table 7:** Results of descriptive analysis and correlation analysis.

Variable	Mean	SD	Median	Self-efficacy	Negative emotion	Positive emotion	Teaching engagement
Self-efficacy	3.843	0.691	3.875	1			
Negative emotion	2.637	0.907	2.500	−0.269***	1		
Positive emotion	4.022	0.812	4.000	0.274***	−0.046	1	
Teaching engagement	3.686	0.777	3.731	0.435***	−0.352***	0.368***	1

***p < 0.001.

Table 7 also showed the relationship between university English teachers’ information-empowered teaching engagement and their emotional experiences. A robust positive correlation between teaching engagement and positive emotion (*r* = 0.368, *p* < 0.001), and a significant negative correlation with their negative emotional experiences (*r* = −0.352, *p* < 0.001) were demonstrated. This indicated that the more positive emotions teachers experienced during the teaching process, the more they engaged in information-based teaching, and vice versa.

### 4.3 Main effect and mediation analysis

#### 4.3.1 Multicollinearity test

Prior to conducting SEM analysis, rigorous diagnostics for multicollinearity among variables was performed to ensure the robustness of the analytical framework. The diagnostic examination included calculating both variance inflation factor (VIF) and tolerance indices for all variables (see Table 8).

**Table 8:** Results of multicollinearity diagnosis.

Variable	VIF	Tolerance
Self-efficacy	1.287	0.777
Negative emotion	1.179	0.848
Positive emotion	1.195	0.837
Teaching engagement	1.469	0.681

Following established methodological guidelines (Hair et al., 2022), VIF less than 10 and tolerance greater than 0.1 indicate no serious multicollinearity problems. In this study, the assessment of multicollinearity revealed that all VIFs ranged from 1.179 to 1.469, well below the conservative cutoff of 10; meanwhile, tolerance values ranged from 0.681 to 0.848, exceeding the threshold of 0.1. These results proved that the linear associations among predictor variables were within acceptable limits and confirmed the absence of substantial multicollinearity. Thus, these results supported the robustness of the subsequent SEM analysis.

#### 4.3.2 Measurement model evaluation

The confirmatory factor analysis (CFA) was employed to examine the reliability and validity of the measurement model. A common latent factor (CLF) was incorporated into CFA. As shown in Table 9, the results demonstrated a good model fit for the measurement model (χ^2^/df = 1.008, RMSEA = 0.006, SRMR = 0.034, GFI = 0.921, AGFI = 0.921, NFI = 0.954, TLI = 0.989, CFI = 0.977, and IFI = 0.989). Most indices met the recommended thresholds, supporting the structural validity of the constructs. Nevertheless, the potential for residual method bias inherent in the cross-sectional self-report design had to be acknowledged as a study limitation.

**Table 9:** CFA model fit indices.

Fit Indices	χ^2^/df	RMSEA	SRMR	GFI	AGFI	NFI	TLI	CFI	IFI
Model results	1.008	0.006	0.034	0.921	0.921	0.954	0.989	0.977	0.989

#### 4.3.3 Structural model and hypothesis testing

**Figure 1:**
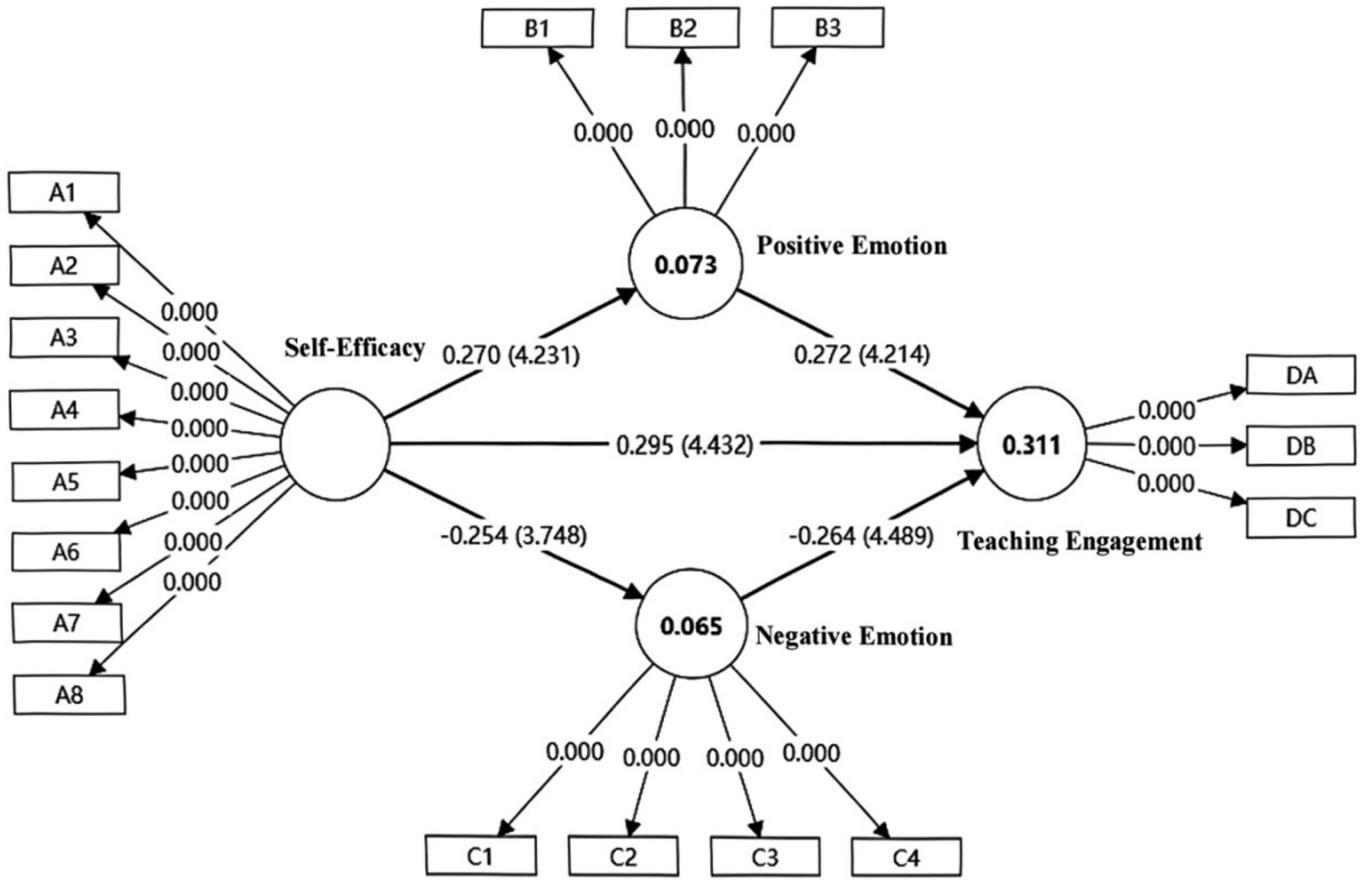
The mediating role of positive and negative emotions between information literacy self-efficacy and information-empowered teaching engagement.

Building upon the validated measurement model, maximum likelihood estimation was used to examine the path coefficients of the structural model. The statistical significance of all paths was verified through Bootstrap method (5,000 samples). Figure 1 shows the mediation model generated by SmartPLS 4 software.

Path analysis revealed that all theoretically hypothesized paths reached statistical significance (see Table 10). Self-efficacy demonstrated a significant positive direct effect on teaching engagement (*β* = 0.295, *t* = 4.432, *p* < 0.05), while simultaneously showing significant negative prediction of negative emotion (*β* = −0.254, *t* = 3.748, *p* < 0.05) and significant positive prediction of positive emotion (*β* = 0.270, *t* = 4.231, *p* < 0.05). Negative emotion exhibited a significant negative effect on teaching engagement (*β* = −0.264, *t* = 4.489, *p* < 0.05), whereas positive emotion demonstrated a significant positive promoting effect on teaching engagement (*β* = 0.272, *t* = 4.214, *p* < 0.05).

**Table 10:** Results of structural model path coefficient analysis.

Path relationships	*β*	SD	*T*-values	*p-*values	95% CI [low/high]	*f* ^2^	*R* ^2^
Self-efficacy → teaching engagement	0.295	0.068	4.432	< 0.05	[0.148, 0.417]	0.109	0.302
Self-efficacy → negative emotion	−0.254	0.068	3.748	< 0.05	[−0.402, −0.139]	0.069	0.060
Self-efficacy → positive emotion	0.270	0.063	4.231	< 0.05	[0.154, 0.399]	0.079	0.069
Negative emotion → teaching engagement	−0.264	0.054	4.489	< 0.05	[−0.383, −0.168]	0.094	
Positive emotion → teaching engagement	0.272	0.065	4.214	< 0.05	[0.145, 0.399]	0.099	

The effect sizes were assessed by the model’s *f*^2^. According to Cohen (1988), values of 0.02, 0.15, and 0.35 represent small, medium, and large effects, respectively. As shown in Table 10, all *f*^2^ values ranged from 0.069 to 0.109, falling within the range of small effects. Notably, the path from self-efficacy to teaching engagement demonstrated the largest effect size (*f*^2^ = 0.109) within the model. Although comparatively small, the effect sizes for the paths from positive emotion (*f*^2^ = 0.099) and negative emotion (*f*^2^ = 0.094) to teaching engagement were very similar, indicating that both emotional pathways contributed to the prediction of teaching engagement with nearly identical strength. Similarly, the effect sizes for the paths from self-efficacy to positive emotion (*f*^2^ = 0.079) and to negative emotion (*f*^2^ = 0.069) were closely aligned.

Finally, the model’s overall explanatory power was assessed by the *R*^2^. The structural model explained 30.2% of the variance in teaching engagement, indicating a substantial prediction of this core outcome. For the mediating variables, the model accounted for 6.9% of the variance in positive emotion and 6.0% in negative emotion. These results established that the model provided a meaningful account of the key constructs, with particularly strong explanatory power for teaching engagement.

#### 4.3.4 Parallel mediation analysis

To deconstruct the mechanisms underlying self-efficacy’s influence, a parallel mediation analysis was conducted (see Table 11). The bootstrap results confirmed a significant total indirect effect, which accounted for 34.8% of the total effect of self-efficacy on teaching engagement. Crucially, the analysis revealed that the two emotional pathways operated with nearly identical effect sizes. The specific indirect effects via both positive and negative emotion were significant and almost equal in magnitude, a finding formally supported by an equality constraint test [Δχ^2^(1) = 0.012, *p* = 0.913]. This pattern demonstrated that the influence of self-efficacy on teaching engagement was partially mediated through two distinct yet equally important emotional channels: the promotion of positive emotions and the alleviation of negative ones.

**Table 11:** Results of parallel mediation analysis.

Effect type	Effect value	SD	95% CI [low/high]	Effect proportion
Direct effect				
Self-efficacy → teaching engagement	0.295	0.068	[0.148, 0.417]	65.2%
Specific indirect effects				
Via positive emotion	0.077	0.024	[0.034, 0.129]	17.5%
Via negative emotion	0.076	0.025	[0.033, 0.129]	17.3%
Total Indirect effect	0.153	0.040	[0.080, 0.235]	34.8%
Total effect	0.440	0.075	[0.293, 0.587]	100%

## 5 Discussion

### 5.1 Emotional experience of university English teachers in information-empowered teaching

Participants in this research have highlighted that incorporating information technology into the classroom has significantly improved university English teachers’ experience of positive emotions. By leveraging instant feedback tools Tecent QQ and WeChat, and online teaching platform like ketangpai,^2^ teachers can foster better student interactions, tailor lessons to individual interests, and adapt their teaching methods to enhance students’ overall learning experience. This has evoked positive emotions of English teachers and a warm reception of technology in English teaching and learning (Liu et al., 2017; Azzaro and Martínez Agudo, 2018). However, Yang et al. (2021) noted a starkly different reaction among primary and secondary school English teachers, who were more skeptical, if not outright negative, about the application of information technology in English teaching.

There are two main reasons for this difference. First, English teaching in university emphasizes the importance of critical thinking, which is consistent with technology convergence goals of college English teaching in China (Guo, 2012), while primary and secondary schools pay more attention to fundamental knowledge and exam preparation, which makes the application of technology inferior to the traditional teaching methods like mechanical vocabulary memorization and grammar drills. Second, according to the Technology Acceptance Model (Davis, 1986), adoption of technology is dependent on perceived convenience and utility. In China, the impetus for English teaching reform often originates from the frontier research of university English teachers. More often than not, primary and secondary English teachers attend programs about information technology and receive training from experts or researchers from universities. In addition, Chinese universities are usually equipped with more abundant teaching resources, providing a broader application scenario for university English teachers. This enables them to be more acutely aware of the convenience and utility of information technology in English teaching, and thus they are more proactive in integrating it into their daily teaching practices.

The statistics of descriptive analysis also indicate that university English teachers experienced negative emotions like anger, disappointment, and anxiety. These emotions typically arose in specific pedagogical contexts, such as when they faced technical glitches or encountered the complexities inherent in educational technology systems. In addition, when teachers struggled with new technologies, they experienced helplessness and frustration. The technological anxiety could be particularly pronounced among older teachers. These findings supplement a series of recent studies on teacher emotions concerning AI. For instance, teachers may experience such negative emotions as fear, frustration and anxiety due to a lack of AI knowledge and literacy, adequate resources to support the use of AI in teaching (Shen and Guo, 2024), and confidence (Ci and Jiang, 2025). Collectively, all these studies, together with the present one, show that technical defects, low information literacy, lack of institutional support and self-efficacy are significant contributors to teachers’ negative emotions.

Therefore, it is necessary for institutions to set up a long term technology support system, carry out specialized training regularly, and update the teaching facilities, so as to reduce the technical burden for teachers. However, it is far from enough for institution administrators to provide technology support only, because a very recent research has pointed out that although professional training can enhance teachers’ technical skills to a certain extent, it does not significantly help them deal with the negative emotions that arise when using technologies such as AI in teaching (Ayanwale et al., 2024). A better solution, as Lo (2023) rightly suggests, is to move beyond purely technical training by implementing institutional supports such as hybrid professional learning communities, blended training with emotionally supportive design, and ongoing coaching. These programmatic levers are essential to amplify teachers’ positive emotions and strengthen their self-efficacy within technology-rich environments.

### 5.2 Self-efficacy in information literacy as a main factor driving engagement in information-empowered English teaching

Statistical analysis reveals a robust correlation between teachers’ information literacy self-efficacy and their teaching engagement. This relationship significantly intensified by the mandatory shift to online teaching during the COVID-19 pandemic (Gobbi et al., 2021). At that time, teachers who possessed a high degree of technological self-efficacy were more likely to be confident in on-line teaching and to embrace information technology as a transformative tool in their classroom teaching practice (Skantz-Åberg et al., 2022). Moving beyond correlation, the heightened self-efficacy serves as a critical psychological resource (Bandura, 1978; Bandura, 1989). It empowers teachers to reframe technology integration from a potential threat into an achievable challenge, thereby reducing the cognitive load and anxiety associated with digital tools. This reduction frees teachers’ mental resources for pedagogical innovation rather than mere technical operation, as evidenced by the increased flexibility in instructional design, classroom management, and the enrichment of student learning experiences observed among confident and experienced teachers (Wang et al., 2021).

Consequently, these teachers are more likely to experiment with advanced tools and refine their instructional strategies, a transformation of engagement from quantitative time investment to qualitative pedagogical exploration. This aligns with the findings of Gong (2023) who affirms that such self-efficacy is instrumental in cultivating innovative techniques. Therefore, enhancing information literacy self-efficacy transcends basic skill training, and it constitutes a foundational psychological intervention that builds teachers’ adaptive capacity and innovative potential. Ultimately, this development is not merely a reaction to technological trends but a crucial pathway to enhancing the efficacy and quality of English education in the digital age.

### 5.3 The mediating role of teacher emotions

In this research, the mediating effect of teacher emotion on the relation between information literacy self-efficacy and the information-empowered teaching engagement is also observed. Path analysis shows that the mediating effects of positive and negative emotions are of comparable strength, suggesting that the absence of negative emotions is just as critical as the presence of positive ones for sustaining teacher engagement in the context of technology integration.

The significant role of positive emotions (e.g., enjoyment, pride) aligns with the broaden-and-build theory (Fredrickson, 2001). Positive emotions like enjoyment and pride broaden teachers’ thought-action repertoires, fuel enthusiasm and foster the exploration and innovation necessary for deep engagement with information-empowered teaching, ultimately leading to the improved teaching quality and more productive learning experience (Romo-Escudero et al., 2023). Conversely, the equally powerful role of negative emotions (e.g., anxiety, frustration) is decisively explained by the control-value theory (Pekrun, 2006). In the rapidly evolving environment of current information-empowered language teaching, a perceived lack of control over technology or doubts about its pedagogical value can trigger potent negative emotions. These emotions are not merely transient feelings but actively constrict cognitive resources, divert attention toward threat management, and can lead to avoidance behaviors, such as a retreat to familiar traditional methods, thereby severely curtailing teaching engagement.

This finding highlights the importance of implementing PP interventions within the socio-cultural context of higher education in East Asian. The emphasis on this context is crucial, because prevailing Western-centric PP approaches, which often prioritize individualistic expression and self-enhancement, may not fully align with the collectivistic values, hierarchical relationships, and nuanced emotional expression, commonly found in many East Asian academic settings. Therefore, responding to the call by Lo and Punzalan (2025), it is important to design culturally responsive programs that integrate technical and emotional support. Such initiatives can be structured around the concept of the emotional bridge (Lo, 2023), which posits that cognitive beliefs are linked to behavioral engagement through emotional states. To institutionalize this connection, programmatic mechanisms should be established, including hybrid professional learning communities, blended training with emotionally supportive design, and ongoing coaching. These mechanisms are vital not only for amplifying positive emotions and strengthening self-efficacy but also for actively intervening to mitigate the impact of negative emotions. By reducing anxiety and frustration through structured and culturally attuned support, such interventions can disrupt the negative emotional pathway, thereby fostering a sustainable and adaptive teaching environment.

## 6 Conclusion

This study, based on the empirical investigation, examines the role of university English teachers’ self-efficacy and emotional experiences in shaping their teaching engagement within information-empowered teaching contexts. The findings demonstrate that enhanced self-efficacy, along with the cultivation of positive emotions, predicts higher levels of teaching engagement. Both positive and negative emotions are found to play equally substantial mediating roles between self-efficacy and teaching engagement. These findings underscore the crucial role of psychological factors in technology-rich teaching environments.

Based on these findings, we recommend that educational institutions including primary, secondary, and higher education establishments should implement a dual support system integrating both technical and psychological assistance. On the one hand, institutions should regularly update teaching facilities and organize technological training; on the other hand, developing hybrid professional development programs incorporating emotional support should be emphasized, so as to intentionally enhance teachers’ positive emotions and effectively alleviate negative emotions. This institutionalized support framework not only helps strengthen teacher self-efficacy but also effectively promotes deep engagement in information-empowered teaching through emotional regulation.

This study has several limitations that should be considered when interpreting the findings. First, the sample was drawn only from Southeast China, and thus the results may not fully represent university English teachers from central or western China where educational resources and teaching environments differ. Second, the gender imbalance in the sample suggests the findings may be more representative of female teacher populations. Future research could employ stratified sampling methods to expand sample coverage and further develop longitudinal or experimental studies to examine the effects of PP interventions on enhancing positive emotions, as well as the roles of hybrid engagement-oriented professional development in the relationship between self-efficacy, emotion, and teaching engagement. This would provide more contextually appropriate theoretical foundations and practical solutions for teacher professional development within East Asian educational contexts.

## Data Availability

The original contributions presented in the study are included in the article/Supplementary material, further inquiries can be directed to the corresponding author.

## Footnotes

1
https://www.wjx.cn/

2
https://jmu.ketangpai.com
