# Supplementary material for: From believing to behaving: Unpacking teacher emotion as the mediator between information literacy self-efficacy and information-empowered teaching engagement
Source: Front Psychol. 2026 Jan 7;16:1696627. doi: 10.3389/fpsyg.2025.1696627 (PMC12819628; doi:10.3389/fpsyg.2025.1696627)
Supplement: Supplementary file 1 [file Supplementary_file_1.docx]

**Appendix 1: Composite Questionnaire of Information Literacy for University English Teachers**

| **Items** | **Factor Loadings** | | | |
| --- | --- | --- | --- | --- |
| **Emotional Experience** | | | | |
| 1. I am glad to see information technology being integrated into English teaching. | 0.845 |  |  |  |
| 3. I am happy that the application of information technology has enhanced students’ learning engagement. | 0.879 |  |  |  |
| 6. I am pleased to find that the use of information technology has enhanced the teaching effect. | 0.863 |  |  |  |
| 2. I feel anxious while using information technology in English classes. |  | 0.780 |  |  |
| 4. I am frustrated that I cannot skillfully operate the information technology teaching platform. |  | 0.810 |  |  |
| 5. I am disappointed by students’ indifferent response to the use of information technology in English teaching. |  | 0.832 |  |  |
| 7. I feel annoyed by the challenge of incorporating information technology into the English teaching activities. |  | 0.774 |  |  |
| **Information literacy self-efficacy** | | | | |
| 1. I can use multimedia equipment, information-based teaching platforms and online resources for lesson preparation and teaching. |  |  | 0.776 |  |
| 2. I can skillfully apply modern information technology to assist students’ English learning. |  |  | 0.799 |  |
| 3. I know where to obtain the resources needed for English teaching. |  |  | 0.782 |  |
| 4. I can use appropriate strategies to search for information. |  |  | 0.821 |  |
| 5. I can regularly update information sources, information technology and information retrieval tools. |  |  | 0.754 |  |
| 6. I can integrate existing and new information to expand the English teaching resource database. |  |  | 0.756 |  |
| 7. I can screen out important information from online resources based on teaching needs. |  |  | 0.842 |  |
| 8. I can accurately judge the validity, reliability and authority of the obtained materials. |  |  | 0.802 |  |
| **Investment in Information Technology-based Teaching** | | | | |
| *Emotional Investment* | | | | |
| 1. I enjoy using the information-based teaching platform for instruction. |  |  |  | 0.804 |
| 2. I am passionate about using the information-based teaching platform for instruction. |  |  |  | 0.817 |
| 3. I really enjoy the process of teaching through information-based teaching platform. |  |  |  | 0.818 |
| *Behavioral Investment* | | | | |
| 1. I am always willing to help colleagues solve problems when they encounter difficulties in operating the information-based teaching platform. |  |  |  | 0.762 |
| 2. I often use social software or the information-based teaching platform to discuss and answer questions with students. |  |  |  | 0.768 |
| 3. I often use online resources for lesson preparation. |  |  |  | 0.849 |
| 4. I often use the information-based teaching platform to conduct English teaching. |  |  |  | 0.774 |
| 5. I often use the information technology-based teaching platform to grade English assignments and tests. |  |  |  | 0.774 |
| *Cognitive Investment* | | | | |
| 1. I make deliberate effort to use information technology in my English teaching. |  |  |  | 0.792 |
| 2. I am always highly focused when using the information-based teaching platform for instruction. |  |  |  | 0.777 |
| 3. I feel time flies when using the information-based teaching platform for instruction. |  |  |  | 0.785 |
| 4. I can persistently overcome the difficulties encountered when using the information-based teaching platform for instruction. |  |  |  | 0.841 |

**Appendix 2: 13-item Information Literacy Self-Efficacy Scale (Atikuzzaman and Zabed Ahmed, 2023)**

1. Use electronic information sources
2. Locate information sources in the library
3. Use library catalogue
4. Locate resources in the library using the library catalogue
5. Select information most appropriate to the information need
6. Synthesize newly gathered information with previous information
7. Interpret the visual information (i.e., graphs, tables, diagrams)
8. Determine the content and form the parts (introduction, conclusion) of a presentation (written, oral)
9. Prepare a bibliography
10. Create bibliographic records and organize the bibliography
11. Create bibliographic records for different kinds of materials (i.e., books, articles, web pages)
12. Learn from my information problem solving experience and improve my information literacy skill
13. Criticize the quality of my information seeking process and its products
